# Parental Self-Perception, Parental Investment, and Early Childhood Developmental Outcomes: Evidence From Rural China

**DOI:** 10.3389/fpubh.2022.820113

**Published:** 2022-03-31

**Authors:** Lei Wang, Ting Wang, Hui Li, Kaiwen Guo, Lynn Hu, Siqi Zhang, Scott Rozelle

**Affiliations:** ^1^International Business School, Shaanxi Normal University, Shaanxi, China; ^2^Shanghai Pinghe School, Shanghai, China; ^3^Pardee RAND Graduate School, Santa Monica, CA, United States; ^4^Stanford Center on China's Economy and Institution, Stanford University, Stanford, CA, United States

**Keywords:** parental self-perception, parental investment, early childhood development, mediation effect, rural China

## Abstract

Using a three-wave longitudinal survey conducted in 815 households in rural Western China, this study aims to examine the association between parental self-perception and early childhood development and the mediation effect of parental investment on the association between parental self-perception and child development when the sample children are at different ages in the early childhood (18–30, 22–36, and 49–65 months). The results demonstrate that parental self-perception are positively and significantly associated with child social-emotional development in all three ages of childhood (from 18 to 65 months). Positive and significant association between parental self-perception and child cognitive development is found in the ages from 22 to 65 months. In addition, findings of this study show that parental investment plays a mediating role in the association between parental self-perception and child cognitive development. The study calls on policymakers to help to strengthen parental self-perception and parental investment related to early childhood development, which should result in better child development in rural China.

## Introduction

Early childhood development (ECD) is vital to and predictive of a child's overall growth and development (1–7). International studies have shown extensive evidence that human brains are vulnerable to various biological, psychosocial, and environmental factors during the first few years of life, and these factors have been shown to be critical in shaping the attributes of individuals, contributing to a variety of lifelong outcomes, including educational attainment, employment, and earnings (3, 4, 8).

A large body of research in recent years has focused on identifying the key factors that influence ECD outcomes (9–12). Many of these studies have found that the intensity and quality of parental investment are associated with ECD (4, 6, 11, 13–18). Specifically, the international literature has demonstrated that parental investment, such as reading, singing songs, and playing interactively with a child, is associated with child cognitive, language, and social-emotional development (18–20). A meta-analysis of 46 studies worldwide found a strong and positive association between parental investment and child outcomes in math, reading, and language domains (21). One study that focused on low-income mothers and their children in the United States (US) found that parental investment (i.e., a variety of parent-child interactions) prior to school entry affected not only the child cognitive outcomes but also their academic achievement (22). A cross-sectional study of 1,431 children aged 36–59 months in the Honduras showed that early psychosocial stimulation was significantly and positively associated with the developmental outcomes of rural children (23). Likewise, a study conducted in a low-income sample of 2,089 children and families in the US found that parenting quality was significantly associated with the cognitive performance of young children. Specifically, parenting quality mediated the effects of family resources on the performance of children during early childhood (14, 24, and 36 months) (17).

Other studies, conducted in developed and developing countries, have found that parental self-perception of parenting also is associated with positive child developmental outcomes (24–26). Parental self-perception of parenting refers to how individuals perceive themselves in their parenting duties and includes several characteristics such as the feeling of competence experienced in the role of parent; the parent's involvement in caregiving; the feeling of satisfaction from caregiving relationship, and the ability to balance parenting with other roles in life (27–30). Parental self-perception has been linked to the concept of self-efficacy, as it evaluates the way parents perceive their own efficacy in performing their parental roles which in turn is able to create powerful predictors of more effective and sensible parental practices (31–33). In the theoretical model developed by Furstenberg et al. (34) and Bandura (32), parents who have higher levels of self-perception are more likely to increase the likelihood of their children's success in both cognitive and social-emotional development (32, 34). The experimental studies provide supportive evidence to this theoretical framework (24, 35–38). Based on data from 90 parent-child dyads (children aged 24–35 months), a study in Germany found an association between parental perception and child cognitive developmental outcomes (39). In particular, the study found that parental perception can predict inhibitory control in toddlers, which is a critical indicator of the developmental level of toddlers and is known to be related to academic achievement, social-emotional competence, and other long-term developmental outcomes.

Studies also have shown that parental self-perception of parenting is a significant predictor of the level of parental investment in their children, through which parental self-perception can have an effect on child developmental outcomes (37, 40–45), working paper. Parents who perceive themselves as competent in their role as parents can be expected to be more positive about their roles and interact with their children in warm and sensitive ways. The more competent the parents feel about themselves in their roles as parents, the more investment, it has been shown, that they make in child caregiving (27, 37). For example, one study conducted in the US found that parental self-perception was positively associated with positive parenting practices and negatively associated with negative parenting practices such as inconsistent discipline and love withdrawal (36). Further, evidence suggests a mediation effect of parental investment, through which parental self-perception can affect their child, which further has been shown to influence their child's developmental outcomes (36, 46). Another study in the US showed that parental self-perception was linked to child developmental outcomes through parental investment. Specifically, mothers with higher levels of self-perception were shown to engage in more positive parenting practices that promote child development (24).

In rural China, cognitive and social-emotional development delays are distressingly common among children under 5 (47–53). In a recent meta-analysis that included 18 empirical studies conducted in rural China, the results indicated that, on average, the rates of cognitive and social-emotional delays among children under 5 were 45 and 36%, respectively (48). Using a large sample (*N* = 3,353) across four main rural populations in China, Wang et al. (50) found that 49% of the sample children (aged 6–30 months) were delayed in cognition and 53% were delayed in social-emotional development (50). Further, a number of empirical studies in different geographical areas of rural China have found that the rates of cognitive and social-emotional developmental delays of children aged 0–3 years were very high, ranging from 39 to 58% (50, 51, 54–61). Studies of preschool-aged children in rural China also found consistently high rates of cognitive and social-emotional delays (62–65). In a study of 505 poor rural children, aged 4 to 5, the proportion of these children with delayed cognitive development was found to be 57% (63). Another study, conducted in rural Jiangsu, found that 37% of sampled preschool children had social-emotional developmental delays (65). In contrast, the rate of delay in urban children (ages 2–36 months) was found to be around 10% (66–68). In comparison, in healthy populations worldwide, rates of developmental delay in infants and toddlers have been found to be ~15%, (Rozelle, 2016, presentation). The rate of developmental delay for urban preschoolers in China has been measured to be around 15%, which is what is expected to be found in a healthy population (69, 70).

Studies of rural China have revealed that low levels of parental investment might be one reason for the high rates of child developmental delays (50, 58, 71–73). One study, which focused on 1,442 randomly-selected toddlers (18–30 months) and their caregivers in rural China, found that low levels of parental investment, such as reading, singing, or engaging in stimulating play with their children, were strongly associated with child cognitive delays (61). A study showed that only a small portion of parents (7–24%) of preschool-aged children engaged in different types of positive parenting practices, which are a main type of parental investment. The authors found that the lack of positive parenting practices was associated with high levels of child cognitive developmental delays at preschool age (38%) (74).

Even though parental self-perception have been demonstrated to have direct and indirect effects on levels of child development, few studies in rural China have examined the role of parental self-perception and their association with child development. To the best of our knowledge, there has been only one study that has investigated the mediation effect of parental investment on the association between parental self-perception and ECD in rural China (53). Zhong et al. (53) found that parental investment significantly mediated the association between parental self-perception and the developmental outcomes of children aged 6–24 months. It should be noted, however, that the authors presented findings on children at an early development stage (<2 years old) and used data collected in only one survey wave (53).

The goal of this study is to examine the association between parental self-perception and child developmental outcomes at different ages of the child in rural China, using a three-wave longitudinal data. To achieve this goal, we have three objectives. First, we describe child developmental outcomes, parental self-perception, and parental investment when children are at different ages (18–30, 22–36, and 49–65 months). Second, we examine the associations between parental self-perception, parental investment, and child developmental outcomes at different ages of the child. Third, we analyze the mediation effect of parental investment on the association between parental self-perception and child developmental outcomes.

To meet these objectives, we used a multistage cluster sampling design and conducted a longitudinal study in 11 nationally designated poverty counties in rural China. The data used in this study were collected in three waves (when children were 18–30, 22–36, and 49–65 months old). All children and their caregivers who participated in all three waves were included (*N* = 815). We measured parental investment and parental self-perception by interviewing caregivers. Child cognitive development was assessed using the Bayley Scales of Infant Development (BSID) for children under 30 months old, the Griffith Mental Development Scales (GMDS-ER) for children between 30 and 36 months old, and the Chinese version of the Wechsler Preschool and Primary Scale of Intelligence-Fourth Edition (WPPSI-IV) for children at preschool age (49–65 months old). Using ordinary least squares (OLS) regression analysis, we examined the associations between parental investment and child developmental outcomes as well as the associations between parental self-perception and child developmental outcomes in the three waves. Following the standard mediation analysis model, we explored the mediation effect of parental investment on the association between parental self-perception and child developmental outcomes during different waves.

The results demonstrate that parental self-perception was positively and significantly associated with child cognitive development when the sample children were aged from 22 to 65 months. Specifically, in the Follow-up 2 survey (when children were 22–36 months old) and the Follow-up 3 survey (when children were 49–65 months old), parental self-perception was positively associated with child cognitive scores by 0.06 SD (*p* < 0.01) and 0.07 SD (*p* < 0.05), respectively. The results also show that parental self-perception was positively and significantly associated with child social-emotional development in all three waves. Specifically, in the three waves, parental self-perception was associated with more favorable child social-emotional scores by 0.22 SD (*p* < 0.01), 0.09 SD (*p* < 0.01), and 0.16 SD (*p* < 0.01), respectively. In addition, the findings indicate that parental investment played a mediating role in the association between parental self-perception and child cognitive developmental outcomes. No mediation effect of parental investment, however, was detected for the association between parental self-perception and child social-emotional developmental outcomes.

This study makes two contributions to the literature. First, this is the first longitudinal study to examine the association between parental self-perception and child developmental outcomes in rural China over a relatively long term. Second, using a standard mediation method, we draw conclusions on the mediation role of parental investment on the association between parental self-perception and child development and, thus, call on policymakers to help to strengthen parental self-perception and parental investment in ECD, which should result in better child development in rural China.

The remainder of this paper is as follows. Section Methods provides the methods that we use, including sample selection, data collection, and statistical approaches. Section Results presents the results. Section Discussion includes the findings, and Section Conclusions concludes.

## Methods

### Ethical Approval

This study received ethical approval from the Stanford University Institutional Review Board (IRB) (Protocol ID 25734), and from the Sichuan University Ethical Review Board (Protocol ID 2013005–01). All participating caregivers gave their consent for both their own and their child's involvement in the study.

### Sampling

The data for this study are drawn from a longitudinal study of children and households conducted in 11 nationally designated poor counties of the Qinba Mountain area, which is a relatively poor area in northwest China. In 2013, the per capita GDP of the study area was US$1,275 (RMB 7,896), lower than the national per capita GDP of US$7,057 (RMB 43,684) (75).

Sample selection for this longitudinal study was conducted in 2013 and followed a multistage cluster sampling design. First, all townships in 11 counties were included, excluding the township in each county that housed the county seat and those townships that did not have any villages with a population at least of 800. These exclusion criteria were chosen to ensure a rural sample and increase the likelihood that sampled villages had a sufficient number of children aged 6–12 months. Following the criteria, 174 townships were included in this study. Next, in each of the townships included, two villages were randomly selected. To meet statistical power requirements, we randomly selected an additional village in chosen townships, and 351 villages were included in this study. Finally, a list of all registered births, provided by local officials in each sample village, was obtained, and all children aged 6–12 months were included in the study. Children were enrolled in two cohorts. The first cohort contained children enrolled in April 2013. A second cohort of children in the target age range was enrolled from the same sample villages in October 2013.

Three follow-up surveys (October 2014, when the sample children were 18–30 months old; April 2015, when the sample children were 22–36 months old; and June 2017, when the sample children were 49–65 months old) were completed after the initial survey was conducted, in 2013. In this study, we used data from the three follow-up surveys, as parental self-perception data were not collected in the initial survey in 2013. For analysis, the final sample included 815 children and their families who participated in all three follow-up surveys.

### Data Collection

In each of the three follow-up surveys, four main blocks of data were collected. The first block of data collected was on parental investment. The second block was on parental self-perception. The third block was on the cognitive and social-emotional development of each sample child. The last block of data was on the demographic characteristics of each child and the household.

#### Parental Investment

All primary caregivers were administered a general parenting-oriented survey to assess the nature of parental investment. The questions in the survey were adapted from two primary sources: the parenting module of the Multiple Indicator Cluster Surveys and the National Survey of Early Childhood Health, developed by the U.S. Centers for Disease Control and Prevention. The primary caregiver of each sample child was asked the following four questions about parental investment: “Did you tell stories to your child yesterday?”; “Did you read books to your child yesterday?”; “Did you sing songs to your child yesterday?”; and “Did you play with your child yesterday?” These questions were chosen as indicators of parental investment based on the findings of studies that show that these three indicators are associated with child development (76–81).

#### Parental Self-Perception

As part of second block of the survey, a questionnaire was administered to the primary caregivers to evaluate their parental self-perception. The questionnaire included 12 items, for example: “I really enjoy being with my child”; “I get along with my child”; “I am annoyed when I am with my child”; “I am nervous (stressed) while I'm with my child”; and “I am always ignored by my child when talking to him or her.” All 12 items are shown in the Supplementary Table 1. Primary caregivers used a 5-point scale (1 = “completely incorrect” to 5 = “completely correct”) to score each item. The total score of the caregiver's self-perception was calculated by summing the item scores. The Cronbach's alpha are 0.63, 0.62, and 0.71 for Follow-up 1 survey, Follow-up 2 survey, and Follow-up 3 survey, respectively, indicating that the questionnaire's internal consistency for the sample for each of the follow-up surveys was adequate (82).

#### Child Cognitive and Social-Emotional Development

In the Follow-up 1 survey (when the sample children were 18–30 months old), all children were assessed using the BSID, a standardized test of cognitive development for children under 30 months (83). The test was formally adapted to the Chinese language and environment in 1992 and scaled according to an urban Chinese sample (84, 85). The BSID produces a mental development index (MDI) that is a measure of memory, habitation, problem solving, early number concepts, generalization, classification, vocalizations, and language (83). In the Chinese version of the BSID, the MDI has an inter-rater reliability of 0.99, a test-retest reliability rate of 0.82, and a parallel-forms reliability of 0.85 (86). The MDI has an expected mean of 100 and an SD of 16. Children with an MDI score below 84 (1 SD) are considered developmentally delayed.

In the Follow-up 2 survey (when the sample children were 22–36 months old), because the BSID is not designed to assess outcomes for children older than 30 months, only children aged 30 months or under (approximately half of the sample) were administered the BSID for this survey. Older children's cognitive development outcomes were assessed using the GMDS-ER (87). The GMDS-ER has been shown to be comparable to the BSID in its assessment of early childhood development (88). The GMDS-ER comprises six subscales: locomotor, personal-social, language (receptive and expressive), hand and eye coordination, performance, and practical reasoning. The reliability of the GMDS-ER has been found to be as high as 0.99 (89), and the reliability of the individual subscales also have high levels of internal consistency, ranging between 0.90 and 0.98 (90). Children with GMDS-ER scores below 85 (1 SD) are considered developmentally delayed.

In the Follow-up 3 survey (when the sample children were 49–65 months old), we assessed cognitive development, using the Chinese version of the WPPSI-IV. The WPPSI-IV is an individually administered, standardized test for assessing the cognitive functioning of children aged 30–91 months (91). The Chinese version of the WPPSI-IV was adapted in 2010 and scaled according to a Chinese sample from urban and rural areas (92) and has since been applied in research across China (93, 94). The WPPSI-IV produces a Full-Scale Intelligence Quotient (FSIQ), which is a composite score that provides a summary of cognitive ability across a diverse set of domains. The FSIQ of Chinese version of the WPPSI-IV test has a reliability coefficient of 0.96. Children with FSIQ scores below 85 (1 SD) are considered developmentally delayed.

All BSID, GMDS-ER, and WPPSI-IV enumerators attended a weeklong training course on how to administer the assessments, including a 2.5-day experiential learning program in the field. The BSID and GMDS-ER tests were administered in the household, and the WPPSI-IV test was administered either in the household or in the school, using a standardized set of toys and a detailed scoring sheet. The primary caregiver or the teacher was required to stay with the child but was not allowed to assist the child during the administration of the tests.

In each of the three follow-up surveys, the children's social-emotional development was assessed using the Ages and Stages Questionnaire: Social Emotional (ASQ: SE) (95). The items in this questionnaire (which vary by age) measure a child's tendency toward a set of behaviors, such as the ability to calm down, accept directions, demonstrate feelings for others (empathy), communicate feelings, initiate social responses to parents and others, and respond without guidance (move to independence). The primary caregiver of each child was asked to indicate whether the child exhibits these behaviors “most of the time,” “sometimes,” or “never.” Depending on the desirability of the behavior, the answers are scored 0, 5, or 10 points. In interpreting the results that reader should be aware that the scale of ASQ: SE is inverted, which means that the higher the score, the lower the level of social-emotional development. In 2017, the ASQ: SE was culturally modified and normalized in China. The Chinese version of the ASQ: SE scale has a test-retest reliability of 0.94, and its item reliability by age ranges from 0.94 to 0.96 (96). Social-emotional delay is defined as having social-emotional scores higher than the cutoff set for different ages by the ASQ: SE manual (Supplementary Table 2).

#### Socioeconomic Survey

Teams of trained enumerators collected child and household characteristics from each sample child's primary caregiver. During the survey, all family members at home were asked who was most responsible for the child's daily caregiving. The individual who was responsible for all (or almost all) caregiving activities of the child was identified as the primary caregiver. Child characteristics included the child's age in months, gender, whether the child was premature (born before 37 weeks of gestation), and whether the child had a low birth weight. Household characteristics included whether the child's mother was the primary caregiver, the primary caregiver's age, the primary caregiver's education level, whether the father migrated for work, whether the mother migrated for work, and whether the family received social security support.

### Statistical Analysis

For our analysis, the raw scores of the BSID, GMDS-ER, WPPSI-IV, and ASQ: SE were standardized separately for each survey wave. Because raw scores increase with age, we computed age-adjusted standardized scores by subtracting age-specific means and dividing by age-specific SDs, estimated using non-parametric regression methods. This method is used mainly because the number of sample observations in each age segment is relatively small, and this procedure makes the data less sensitive to outliers (97). Using this approach yields normally distributed standardized scores with a mean of zero across the age range. In addition, the scores for parental self-perception and parental investment used in the regression are factor analyzed and standardized.

To examine the associations between parental investment and child developmental outcomes at the three follow-up surveys, we employed OLS to construct a model as follows:


(1) $$\begin{array}{l} \mathrm{Developmen}t_{i}=\alpha +{\beta \;*\;\mathrm{Investment}}_{i}+\gamma X_{i}+\varepsilon_{i} \end{array}$$

where the dependent variable, *Development*_*i*_, is the standardized score of child cognitive or social-emotional development at the three different surveys. The variable *Investment*_*i*_ represents the factor *z*-score of parental investment of child *i* for the three different surveys. *X*_*i*_ is a vector of covariates that capture demographic characteristics, including the child's age (in months) and gender, whether the child was premature (born before 37 weeks of gestation), whether the child had a low birth weight, whether the child's mother was the primary caregiver, the primary caregiver's age, the primary caregiver's education level, whether the father migrated for work, whether the mother migrated for work, and whether the family received social security support. ε_*i*_ is an error term. The analysis also accounted for clustering at the village level.

We also used the same OLS regression approach with an alternative specification to estimate the associations between parental self-perception and child developmental outcomes. The model is constructed as follows:


(2) $$\begin{array}{l} \mathrm{Developmen}t_{i}=\alpha \;+\;{\beta \;*\;\mathrm{Selfperception}}_{i}\;+\;\gamma X_{i}\;+\;\varepsilon_{i} \end{array}$$

where the variable *Selfperception*_*i*_ represents the factor z-score of parental self-perception of child *i*. The dependent variable *Development*_*i*_ and the terms *X*_*i*_ are defined as above in equation (1). The analysis also accounted for clustering at the village level.

To examine the association between the parental investment and parental self-perception, we employed the same OLS estimation approach as used in equations (1) and (2). We used the following model:


(3) $$\begin{array}{l} \mathrm{Investmen}t_{i}=\alpha \;+\;{\beta \;*\;\mathrm{Selfperception}}_{i}\;+\;\gamma X_{i}\;+\;\varepsilon_{i} \end{array}$$

Equation (3) is similar to equation (2), except that the independent variable is transposed to *Investment*_*i*_, which represents the factor z-score of parental investment of child *i*.

To estimate the mediation effect of parental investment on the association between parental self-perception and child developmental outcomes (that is, to investigate to what extent the effect of parental self-perception on child development can be explained by the channel of the effect of parental self-perception on parental investment), we employed the following mediation model:


(4) $$\begin{array}{l} \mathrm{Developmen}t_{i}=\alpha +{\beta_{1}\;*\;\mathrm{Selfperception}}_{i}+\beta_{2}\;{*\;\mathrm{Investment}}_{i}\\ \;+\gamma X_{i}+\varepsilon_{i} \end{array}$$

In this equation, the notation used is analogous to the notation in equations (1), (2), and (3). The mediator is parental investment. Following Preacher and Hayes (98), standard errors (SE) of the indirect effects were computed using the bootstrap method, based on resampling 1,000 replications. One type of 95% confidence interval (CI), i.e., a bias-corrected (BC) interval, also was calculated to test the statistical significance of the indirect effects (98).

## Results

### Demographic and Socioeconomic Characteristics

Table 1 presents the demographic and socioeconomic characteristics of the sample children and their caregivers for Follow-up 1. Of the 815 children in this study for the Follow-up 1 survey, the average age was 24 months, and slightly over half (51%) were male. Only a small proportion of the children (4%) were born prematurely, and about 5% of the children had low birth weight. With regard to the household characteristics of the sample respondents, 64% of the children had their mothers as their primary caregivers (most of the remaining children had their grandmothers as their primary caregivers). The average age of the primary caregiver was 32 years. About one-third (34%) of the primary caregivers did not complete 9 years of schooling. The results also indicate that, for 32% of the sample children, their mothers out migrated for work, and in the case of over half of the sample children, their fathers out migrated for work (58%). In addition, a quarter (25%) of the sampled households received the Minimum Living Standard Guarantee Payments, a form of government welfare for rural China's lowest-income families.

**Table 1:** Characteristics of child and household (18–30 months) (*N* = 815).

**Characteristic**	**Mean (SD)**
**Child**	
Age in months	23.70 (3.17)
Male (1 = yes)	0.51 (0.50)
Premature (1 = yes)	0.04 (0.21)
Low birth weight (1 = yes)	0.05 (0.21)
**Household**	
Primary caregiver (1 = mother)	0.64 (0.48)
Caregiver age (years)	31.94 (10.36)
Caregiver education (1 = below 9 years)	0.34 (0.47)
Mother migrates for work (1 = yes)	0.32 (0.46)
Father migrates for work (1 = yes)	0.58 (0.49)
Household receives social security (1 = yes)	0.25 (0.43)

### Parental Investment, Parental Self-Perception, and Child Developmental Outcomes

Parental investment, parental self-perception, and child developmental outcomes are reported in Table 2 for all three survey waves (when children were 18–30, 22–36, and 49–65 months old). The data show that a large share of the children experienced cognitive and social-emotional delay. We found that 46% of the children in the Follow-up 1 survey (18–30 months), 42% in the Follow-up 2 survey (22–36 months), and 41% in the Follow-up 3 survey (49–65 months) were cognitively delayed. More than half of the children were social-emotionally delayed. The rates of social-emotional delay were 55, 60, and 53% for the three surveys, respectively.

**Table 2:** Child developmental outcomes, parental investment, and parental self-perception (*N* = 815).

**Variable**	**Follow-up 1 (18–30 months) Mean (SD)**	**Follow-up 2 (22–36 months) Mean (SD)**	**Follow-up 3 (49–65 months) Mean (SD)**	**Difference (1)–(2) *p*-value**	**Difference (1)–(3) *p*-value**	**Difference (2)–(3) *p*-value**
	**(1)**	**(2)**	**(3)**	**(4)**	**(5)**	**(6)**
**Child developmental outcomes**						
Cognitive delay (1 = yes)	0.46 (0.50)	0.42 (0.49)	0.41 (0.49)	0.162	0.064	0.651
Social-emotional delay (1 = yes)	0.55 (0.50)	0.60 (0.49)	0.53 (0.50)	0.072	0.297	0.004
**Parental investment**						
Total parental investment score	0.91 (1.04)	1.10 (1.21)	0.67 (0.97)	0.001	0.000	0.000
Told story to the child yesterday (1 = yes)	0.13 (0.33)	0.20 (0.40)	0.14 (0.35)	0.000	0.344	0.004
Read book to the child yesterday (1 = yes)	0.04 (0.20)	0.10 (0.30)	0.07 (0.25)	0.000	0.036	0.007
Sang song to the child yesterday (1 = yes)	0.37 (0.48)	0.40 (0.49)	0.22 (0.42)	0.127	0.000	0.000
Played with the child yesterday (1 = yes)	0.38 (0.49)	0.40 (0.49)	0.24 (0.43)	0.388	0.000	0.000
**Parental self-perception**						
Total parental self-perception score	47.66 (6.34)	47.46 (6.28)	48.59 (6.04)	0.535	0.002	0.000

*The total parental self-perception score is calculated by summing the scores of the 12 items. The total parental investment score is calculated by summing the scores of the 4 items*.

The empirical data also show that only a small share of primary caregivers engaged in the four types of parental investment that were covered in the survey. Specifically, 13, 20, and 14% of primary caregivers had told a story to their child during the days prior to the three follow-up surveys, respectively. In the case of the parental investment in reading books to their child during the days prior to the surveys, the prevalence was 4, 10, and 7% for the three follow-up surveys, respectively. Likewise, 37, 40, and 22% of primary caregivers indicated that they had sung to their child, and 38, 40, and 24% reported that they had played with their child during the days prior to the three surveys, respectively.

The results for parental self-perception indicate that, on average, the total parental self-perception scores were 47.66, 47.46, and 48.59 for the three surveys, respectively. It is worth noting that the range of the total parental self-perception score was 12–60, which was calculated by summing the scores of the 12 items. Supplementary Table 1 includes the scores of individual items of parental self-perception for three follow-up surveys.

### Associations of Parental Investment, Parental Self-Perception, and Child Development

Table 3 presents the associations between parental investment and child developmental outcomes. The findings indicate that parental investment was significantly correlated with child developmental outcomes. Specifically, in each of the three follow-up surveys, parental investment factor z-scores were positively and significantly associated with child cognitive developmental outcomes. In the case of the social-emotional developmental outcomes of the children, significant and negative associations with the parental investment variables were found in the first two follow-up surveys (note that the scale of the social-emotional development is inverted, meaning the higher the score, the lower the level of social development). No significant association between paternal investment and child social-emotional development was found in the third follow-up survey (when the sample children were 49–65 months old).

**Table 3:** Associations between parental self-perception, parental investments and child developmental outcomes at different surveys (*N* = 815).

	**Follow-up 1** **(18–30 months)**		**Follow-up 2** **(22–36 months)**		**Follow-up 3** **(49–65 months)**	
**Variable**	**Standardized** **cognitive score**	**Standardized** **social-emotional score**	**Standardized** **cognitive score**	**Standardized** **social-emotional score**	**Standardized** **cognitive score**	**Standardized** **social-emotional score**
	**(1)**	**(2)**	**(3)**	**(4)**	**(5)**	**(6)**
**Parental investment**						
Parental investment factor *z*-score	0.10***	−0.08**	0.11***	−0.07**	0.07**	0.02
	(0.03)	(0.03)	(0.03)	(0.03)	(0.03)	(0.04)
Control variables	Yes	Yes	Yes	Yes	Yes	Yes
*R*-squared	0.05	0.03	0.15	0.10	0.19	0.08
Parental self-perception						
Parental self-perception factor z-score	0.06	−0.21***	0.07**	−0.09***	0.08**	−0.16***
	(0.04)	(0.04)	(0.03)	(0.03)	(0.03)	(0.04)
Control variables	Yes	Yes	Yes	Yes	Yes	Yes
*R*-squared	0.05	0.07	0.14	0.11	0.19	0.11

*Control variables include the child's age, gender, whether the child was born prematurely, whether the child had a low birth weight, whether the mother was the primary caregiver, caregiver's age and educational level, whether the mother migrated for work, whether the father migrated for work, and whether the household received social security support. In addition, in Follow-up 2 and Follow-up 3 regressions, the outcome variables in the prior survey (i.e., child developmental outcomes: cognition and social-emotional) also are controlled for in the previous survey for Columns 3–6). All standard errors, in parentheses, are clustered at the village level.*

**p < 0.05, ***p < 0.01.

Table 3 also presents the results of the analysis of the association between parental self-perception and child development. The results indicate that parental self-perception factor z-scores were positively and significantly associated with child standardized cognitive scores in the last two follow-up surveys (when the sample children were 22–36 months and 49–65 months old, respectively). In particular, a 1-SD increase in the parental self-perception factor z-score was associated with a 0.07-SD increase in the standardized cognitive score in the Follow-up 2 survey (when the sample children were 22–36 months) and a 0.08-SD increase in the Follow-up 3 survey (when the sample children were 49–65 months) (*p*s < 0.05). For the first follow-up survey, when the sample children were 18–30 months old, no significant association was found between parental self-perception and child cognitive development.

Finally, negative and significant associations between parental self-perception and child social-emotional developmental outcomes were found for all three surveys. Specifically, a 1-SD increase in the parental self-perception factor z-scores were associated with a 0.21-SD, 0.09-SD, and 0.16-SD decrease in the standardized social-emotional score in the three follow-up surveys, respectively (*p*s < 0.01).

### Mediation Effects of Parental Investment on the Association Between Parental Self-Perception and Child Developmental Outcomes

Table 4 presents the estimates of the associations between parental self-perception, parental investment, and child developmental outcomes in the three follow-up surveys. After we controlled for parental investment, the association between parental self-perception and child standardized cognitive score remained significantly positive (*p* < 0.05) in the Follow-up 3 survey. In contrast, no significant associations were found between parental self-perception and the child standardized cognitive scores in the cases of the first two follow-up surveys. In addition, parental investment was significantly and positively associated with child cognitive development at the Follow-up 1 survey (*p* < 0.01), Follow-up 2 survey (*p* < 0.01), and Follow-up 3 survey (*p* < 0.05). The associations between parental self-perception and child social-emotional development remained significant (*p*s < 0.01) in all three follow-up surveys, after we controlled for parental investment. The coefficient on the parental self-perception factor z-score variable was positively and significantly different from zero, showing a significant association between parental self-perception and parental investment. Specifically, a 1-SD increase in the parental self-perception factor z-score was correlated with 0.08-SD (*p* < 0.05), 0.18-SD (*p* < 0.01), and 0.08-SD (*p* < 0.05) increases in the parental investment factor z-score for the three follow-up surveys, respectively. The results in Table 4 indicate that the mediating effects of parental investment appear in the association between parental self-perception and child developmental outcomes.

**Table 4:** Associations between parental self-perception, parental investment and child developmental outcomes (*N* = 815).

**Variable**	**Standardized cognitive score**	**Standardized social-emotional score**	**Parental investment factor *z*-score**
	**(1)**	**(2)**	**(3)**
Follow-up 1 (18–30 months)			
Parental self-perception factor *z*-score	0.05	−0.21***	0.08**
	(0.04)	(0.04)	(0.03)
Parental investment factor *z*-score	0.09***	−0.06	
	(0.03)	(0.03)	
*R*-squared	0.05	0.07	0.01
Follow-up 2 (22–36 months)			
Parental self-perception factor z-score	0.05	−0.08***	0.18***
	(0.03)	(0.03)	(0.03)
Parental investment factor *z*-score	0.10***	−0.05	
	(0.03)	(0.03)	
*R*-squared	0.15	0.10	0.10
Follow-up 3 (49–65 months)			
Parental self-perception factor *z*-score	0.07**	−0.17***	0.08**
	(0.03)	(0.04)	(0.03)
Parental investment factor *z*-score	0.07**	0.03	
	(0.03)	(0.04)	
*R*-squared	0.20	0.11	0.06
Control variables	Yes	Yes	Yes

*Control variables include the child's age, gender, premature birth, whether the child had a low birth weight, whether mother was the primary caregiver, caregiver's age and educational level, whether the mother migrated for work, whether the father migrated for work, and whether the household received social security support. In addition, in the Follow-up 2 and Follow-up 3 regressions, the outcome variables in the prior survey (i.e., child developmental outcomes: cognition and social-emotional) also were controlled for (in the previous survey for Columns 1 and 2; parental investment in the previous survey for Column 3). All standard errors, in parentheses, are clustered at the village level.*

^**^p < 0.05, ^***^p < 0.01.

Table 5 provides the estimates of the indirect effects of parental self-perception on child cognitive and social-emotional development through parental investment. In the case of child cognitive development, the results show that the point estimates were positive, and the 95% CI (BC) did not overlap with zero in any of the three follow-up surveys. Specifically, the indirect effects of parental self-perception on child cognitive development through parental investment were statistically significant. In the case of child social-emotional development, significant and statistical indirect effect of parental self-perception through parental investment was found only in the Follow-up 1 survey. No effects, however, were found in the Follow-up 2 and Follow-up 3 surveys.

**Table 5:** Estimates of indirect effects of parental self-perception on child developmental outcomes through parental investment.

**Indirect effects**	**Point Estimates**	**Bootstrap S.E**.	**95% CI (BC)**
	**(1)**	**(2)**	**(3)**
Follow-up 1 (18–30 months)			
Parental self-perception on child cognition though parental investment	0.007	0.004	(0.001, 0.017)
Parental self-perception on child social-emotional development through parental investment	−0.005	0.003	(-0.014,−0.0003)
Follow-up 2 (22–36 months)			
Parental self-perception on child cognition though parental investment	0.018	0.006	(0.006, 0.031)
Parental self-perception on child social-emotional development through parental investment	−0.009	0.006	(-0.023, 0.001)
Follow-up 3 (49–65 months)			
Parental self-perception on child cognition though parental investment	0.006	0.004	(0.0003, 0.016)
Parental self-perception on child social-emotional development through parental investment	0.003	0.004	(-0.002, 0.013)

*Bootstrap standard errors reported in Column 2 are based on resampling with 1,000 replications. Column 3 reports one type of 95% confidence interval (CI), i.e., a bias-corrected (BC) interval, and it corrects for a bias in the distribution of bootstrap estimates of standard error*.

As a robustness check, we also used a path analysis method to analyze the associations between parental self-perception, parental investment and child developmental outcomes. The results of the path analysis are the same as the results from the mediation model (see Supplementary Figure 1).

## Discussion

Using data from three survey waves that followed children from 18–30 to 22–36 to 49–65 months, we examined the association between parental self-perception and child developmental outcomes in rural China. We also explored the mediation effects of parental investment on the association between parental self-perception and child developmental outcomes. Our findings demonstrated that parental self-perception was positively and significantly associated with child social-emotional development in all three survey waves. Positive and significant association between parental self-perception and child cognitive development was found in the last two survey waves. The analysis also showed that parental investment significantly mediated the association between parental self-perception and child developmental outcomes.

This study first showed the high prevalence of developmental delays among the sample children in all three survey waves (Follow-up 1, Follow-up 2, and Follow-up 3 surveys). Rates of delay are higher than what one would expect for children in a healthy population [15%, (88)]. Specifically, 46% (when the children were 18–30 months), 42% (when the children were 22–36 months), and 41% (when the children were 49–65 months) of the children experienced cognitive delays. In the same survey waves, 55, 60, and 53% of the children, respectively, experienced social-emotional development delays. These high levels of developmental delays found in this study are consistent with findings from recent studies in rural China (50, 51, 54, 55, 57–61). The results of those studies indicate that cognitive and social-emotional development delays in children before the age of 5 is a common problem in rural China.

Our data also indicated that parental investment was poor in rural China. Overall, less than one-fifth of the primary caregivers engaged in storytelling or book reading to their children, and less than two-fifths of the primary caregivers sang songs to or played with their children. Our findings in regard to poor parental investment of rural primary caregivers in China also are consistent with the results of the previous studies (50, 58, 73). According to these studies, primary caregivers rarely make such a parental investment in rural China.

The findings also showed that parental investment was positively and significantly associated with child cognitive and social-emotional outcomes when we used data from any of the three follow-up surveys (or survey waves). In other words, children who received more parental investment were more likely to reach their developmental potential (or have lower rates of development delays) than were children who received less of such an investment. These results are similar to those of related research (4, 6, 13–16, 21–23). Based on these studies, low levels of parental investment in their children are strongly associated with poor child developmental outcomes.

The findings of the analysis also showed that parental self-perception was positively and significantly associated with child developmental outcomes, which also is consistent with previous studies (24, 36–38, 99). According to these studies, children whose parents have higher parental self-perception scores have more favorable levels of development. Our study, therefore, can be viewed as contributing to the literature by providing strong statistical evidence of the association between parental self-perception and child cognitive and social-emotional developmental outcomes in rural China. The data from our study also show that parental self-perception is significantly and positively associated with parental investment. These findings are consistent with international research that shows that parental self-perception is a key driver of parental investment (44, 45, 100).

The results of the mediation analysis illustrated that parental investment appears to act as a mediator in the association between parental self-perception and child developmental outcomes. This means that parental investment is a channel through which parental self-perception can be seen to be affecting child development. That is, primary caregivers who had high levels of parental self-perception were those who invested more in their children, and this relationship was shown to be associated with better developmental outcomes of the child. Although there has been only limited work in this specific area, this finding is consistent with the results of earlier studies (43, 46, 53, 101). For example, Zhong et al. (53) found a significant mediation effect of parental investment on the association between parental self-perception and child developmental outcomes in rural China (53). According to the literature, parental self-perception is thought to be an important factor contributing to developmental changes in children. As a result, parents who have strong parental self-perception are those that have better knowledge of appropriate parenting behavior and more confidence in one's own abilities to perform them (18, 22, 39). Previous studies have also shown that parental self-perception improve the quantity and quality of parental investment due to their sensitivity to their child's demands and desires (99, 102, 103). Our findings from the mediation analysis suggested that parental investments can explain a portion of the parental self-perception effect on child cognitive development in all three surveys. However, the analysis also illustrated that it plays only a limited role in mediating parental self-perception effect on child social-emotional development. Among all three surveys, parental investment explained about 12, 26, and 7% of the total effect of parental self-perception on child cognitive development respectively. At the same time, it only explained around 2% of the total effect on child social-emotional development in the Follow-up 1 survey.

Our findings also indicated that the mediating effect of parental investment on child social-emotional development was only significant when children were 18–30 months old. According to McCall's model, it is not until after 18 months that environmental and organismic differences tend to exert a significant impact on child development (104). Previous studies have found that, due to opportunities for experiencing relatively high levels of emotional arousal during parent-child interactions during the first 3 years of life, parents play a particularly important role in shaping a child's social-emotional development (105, 106). Studies also have shown that parent-child interactions can play the role of opening the child to the outside world, which in turn can increase a child's social-emotional skills (107, 108). Because of these interactions, it could explain why the mediating effect on child social-emotional development is only significant in the first round (when children were 18–30 months old).

## Conclusions

We examined the association between parental self-perception and child developmental outcomes at three different ages of the child in rural China. Drawing on longitudinal data from 815 children and their primary caregivers, we found that cognitive and social-emotional development delays were distressingly common among the sample children, and parental investment was poor. The findings also illustrate that parental self-perception was positively and significantly associated with child social-emotional development in all three survey waves. Positive and significant association between parental self-perception and child cognitive development was found in the last two survey waves. Further, parental investment played a mediating role in the association between parental self-perception and child cognitive development. Such results suggest that policymakers need to take action to improve the child development environment in rural China. Programs designed to elevate parental self-perception and programs that aim to improve child development could be an effective approach. An alternative approach may be to implement parental training programs that seek to improve parental investment, which, in turn, could improve child development.

We acknowledge four limitations of this study. First, although this study measures correlations between parental self-perception and child developmental outcomes, causal conclusions cannot be drawn. Clearly, there is a need to conduct a causal study in future research. Second, although the sample families who were part of this study were randomly selected from rural Western China, our sample did not include rural households in Central and Eastern China. The sample also did not include families who live in county seats and towns. Hence, there is a need for further research to be conducted in these other areas that are home to a large share of China's rural population. Third, although the study focused on the quantity of the parental investment (i.e., parent-child interactions), the research team did not measure the quality of the parental investment. Future research should examine the importance of both the quantity and quality of the parental investment in terms of the sources of delays in early childhood development. In addition, this study only examines the associations between parental self-perception, investment and child developmental outcome. The study specifically does not consider the malleability of parental self-perception and their impact on parental investment. Future work should investigate the bidirectional associations between parental self-perception, investment and child development.

## Data Availability Statement

The original contributions presented in the study are included in the article/Supplementary Material, further inquiries can be directed to the corresponding author/s.

## Author Contributions

SR and LW designed the study and contributed in editing of the manuscript. LW and SZ were responsible for data collection. SZ, TW, HL, and LW analyzed and interpreted the data. LW, SZ, TW, KG, and HL drafted the initial manuscript. All authors have read and approved the final manuscript for submission.

## Conflict of Interest

The authors declare that the research was conducted in the absence of any commercial or financial relationships that could be construed as a potential conflict of interest.

## Publisher's Note

All claims expressed in this article are solely those of the authors and do not necessarily represent those of their affiliated organizations, or those of the publisher, the editors and the reviewers. Any product that may be evaluated in this article, or claim that may be made by its manufacturer, is not guaranteed or endorsed by the publisher.
