# Supplementary material for: Parental Self-Perception, Parental Investment, and Early Childhood Developmental Outcomes: Evidence From Rural China
Source: Front Public Health. 2022 Mar 31;10:820113. doi: 10.3389/fpubh.2022.820113 (PMC9008586; doi:10.3389/fpubh.2022.820113)
Supplement: Supplementary file 1 [file Data_Sheet_1.pdf]

Supplementary Material

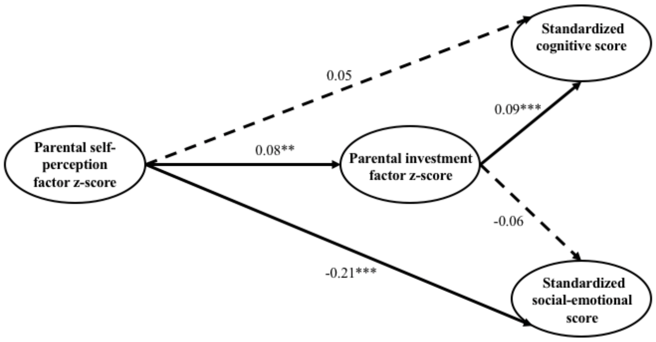

Panel A. Follow-up 1 (18-30 months)

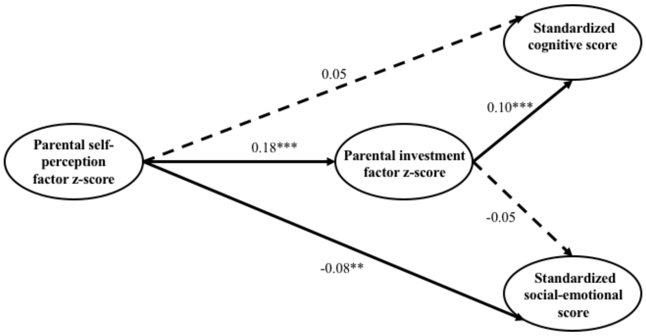

Panel B. Follow-up 2 (22-36 months)

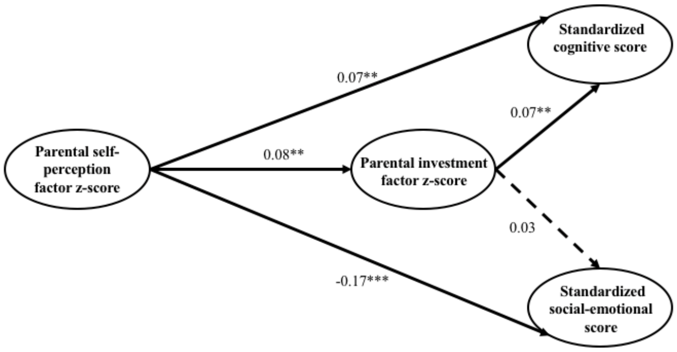

Panel C. Follow-up 3 (49-36 months)

Supplementary Figure 1. Results of the path analysis

Note. The bold lines represent the significant path coefficients and broken lines nonsignificant. Control variables include the child's age, gender, premature birth, whether the child had a low birth weight, whether mother was the primary caregiver, caregiver's age and educational level, whether the mother migrated for work, whether the father migrated for work, and whether the household received social security support. **$p < 0.05$, ***$p < 0.01$

Supplementary Table 1. Parental self-perception items ($N = 815$)

Variable	Follow-up 1 (18–30 months) Mean (SD)	Follow-up 2(22–36 months) Mean (SD)	Follow-up 3(49–65 months) Mean (SD)
	(1)	(2)	(3)
Total parental self-perception score	47.66 (6.34)	47.46 (6.28)	48.59 (6.04)
Item			
I really enjoy being with my child.	4.45 (0.97)	4.43 (1.03)	4.33 (0.87)
I get along with my child.	4.55 (0.80)	4.44 (0.94)	4.37 (0.85)
I am annoyed when I am with my child.	4.21 (1.08)	4.17 (1.06)	4.26 (0.89)
I am nervous (stressed) while I'm with my child.	3.91 (1.39)	4.09 (1.32)	4.37 (1.01)
I am always ignored by my child when talking to him or her.	3.77 (1.32)	3.73 (1.30)	3.80 (1.06)
I do not know how to communicate with my child from his or her perspective.	3.18 (1.54)	3.13 (1.53)	3.26 (1.25)
I think it is fun to play games with my child.	4.33 (1.05)	4.19 (1.11)	3.98 (1.13)
I think playing with my child is important.	4.06 (0.99)	4.06 (1.02)	4.38 (0.81)
I know how to play with my child.	3.86 (1.28)	3.69 (1.33)	3.67 (1.16)
I think reading books or telling stories to my child is important.	3.48 (1.27)	3.56 (1.26)	4.16 (0.91)
I know how to read a storybook with my child.	3.17 (1.53)	3.24 (1.52)	3.41 (1.32)
It is parents' and caregivers' responsibility to help the child understand the world around him or her.	4.70 (0.79)	4.74 (0.67)	4.59 (0.78)

Note. The questionnaire uses a 5-point scale (1 = "completely incorrect" to 5 = "completely correct") to score each item. The total score of the caregiver's parental self-perception was calculated by summing the item scores.

Supplementary Table 2. Applicable range and cutoff score of each ASQ:SE questionnaire interval

Questionnaire interval	Applicable range	Cutoff score
6 months	3–8 months	45
12 months	9–14 months	48
18 months	15–20 months	50
24 months	21–26 months	50
30 months	27–32 months	57
36 months	33–41 months	59
48 months	42–53 months	70
60 months	54–65 months	70

Note. The cutoffs are from the manual of ASQ:SE.
